# Trends of Repetitive Transcranial Magnetic Stimulation From 2009 to 2018: A Bibliometric Analysis

**DOI:** 10.3389/fnins.2020.00106

**Published:** 2020-02-26

**Authors:** Kang-Yong Zheng, Guang-Yan Dai, Yue Lan, Xue-Qiang Wang

**Affiliations:** ^1^Department of Sport Rehabilitation, Shanghai University of Sport, Shanghai, China; ^2^The Fifth Clinical College, Guangzhou Medical University, Guangzhou, China; ^3^Department of Rehabilitation Medicine, The First Affiliated Hospital, Sun Yat-sen University, Guangzhou, China; ^4^Department of Rehabilitation Medicine, Guangzhou First People's Hospital, School of Medicine, South China University of Technology, Guangzhou, China; ^5^Department of Rehabilitation Medicine, Shanghai Shangti Orthopaedic Hospital, Shanghai, China

**Keywords:** rTMS, frontiers, bibliometrics, citation burst, Web of Science, CiteSpace

## Abstract

Repetitive transcranial magnetic stimulation (rTMS) technology, which is amongst the most used non-invasive brain stimulation techniques currently available, has developed rapidly from 2009 to 2018. However, reports on the trends of rTMS using bibliometric analysis are rare. The goal of the present bibliometric analysis is to analyze and visualize the trends of rTMS, including general (publication patterns) and emerging trends (research frontiers), over the last 10 years by using the visual analytic tool CiteSpace V. Publications related to rTMS from 2009 to 2018 were retrieved from the Web of Science (WoS) database, including 2,986 peer-reviewed articles/reviews. Active authors, journals, institutions, and countries were identified by WoS and visualized by CiteSpace V, which could also detect burst changes to identify emerging trends. GraphPad Prism 8 was used to analyze the time trend of annual publication outputs. The USA ranked first in this field. Pascual-Leone A (author A), Fitzgerald PB (author B), George MS (author C), Lefaucheur JP (author D), and Fregni F (author E) made great contributions to this field of study. The most prolific institution to publish rTMS-related publications in the last decade was the University of Toronto. The journal *Brain Stimulation* published most papers. Lefaucheur et al.'s paper in 2014, and the keyword “sham controlled trial” showed the strongest citation bursts by the end of 2018, which indicates increased attention to the underlying work, thereby indicating the research frontiers. This study reveals the publication patterns and emerging trends of rTMS based on the records published from 2009 to 2018. The insights obtained have reference values for the future research and application of rTMS.

## Introduction

Repetitive transcranial magnetic stimulation (rTMS) is a variant of transcranial magnetic stimulation (TMS) that can be applied to the modulation of corticospinal excitability from outside the skull via a time-varying magnetic field to generate electric current in the underlying brain tissue, leading to neuronal depolarization (Maeda et al., 2000; Klooster et al., 2016; Barker and Shields, 2017). rTMS is the most widely used non-invasive brain stimulation technique currently available (George and Aston-Jones, 2010; Miniussi et al., 2013; Cirillo et al., 2017; Lowe et al., 2017; Lucena et al., 2019). Numerous studies have investigated the effects and mechanisms underlying various rTMS protocols, which remain incompletely understood (Fitzgerald et al., 2006b; Boonzaier et al., 2018; Zorzo et al., 2019). Low-frequency (≤1.0 Hz) rTMS usually reduces cortical excitability, whereas high-frequency (>1.0 Hz) rTMS (HF-rTMS) raises excitability (Maeda et al., 2000; Rossini et al., 2015). Over the last decade, rTMS has been explored as a tool for the treatment of various neuropsychiatric conditions, including, but not limited to, depression, neuropathic pain, stroke, epilepsy, anxiety, schizophrenia, Parkinson's disease, obsessive compulsive disorder, and autism (Pascual-Leone et al., 1996; Hummel and Cohen, 2006; Hao et al., 2013; Hosomi et al., 2013). Combined strategies, i.e., combination of rTMS with neuroimaging techniques, medication, physiotherapy, or psychotherapy, have also been investigated to optimize the use of the technique (Reithler et al., 2011; Dayan et al., 2013; Kwon et al., 2016; Jansen et al., 2019; Terranova et al., 2019).

Bibliometrics is a quantitative method for analyzing literature in analytical science and assessing trends in research activities over time (Oelrich et al., 2007; Ellegaard and Wallin, 2015; Thompson and Walker, 2015). Bibliometric studies have been used in various areas, such as medical big data, pain, cognitive function, and neuroimaging, in recent years (Yeung et al., 2017; Liao et al., 2018; Wang et al., 2019; Zheng and Wang, 2019). A considerable number of scholars and academic journals have focused on publishing rTMS research over the last decade. However, reports of trends of rTMS using bibliometric analysis are rare.

This study conducts a bibliometric analysis of rTMS on the basis of records published from 2009 to 2018 to identify the publication patterns and emerging trends of this technique and gain new insights to guide future research and application.

## Materials and Methods

### Source of Data and Search Strategy

Published papers were retrieved via a topic search of the Science Citation Index Expanded (SCI-EXPANDED) index of the WoS database on 6 April 2019. The following search terms were used: topic = (“repetitive transcranial magnetic stimulation” or “rTMS”), index = SCI-EXPANDED and time span = 2009–2018.

### Inclusion Criteria

**Figure 1:**
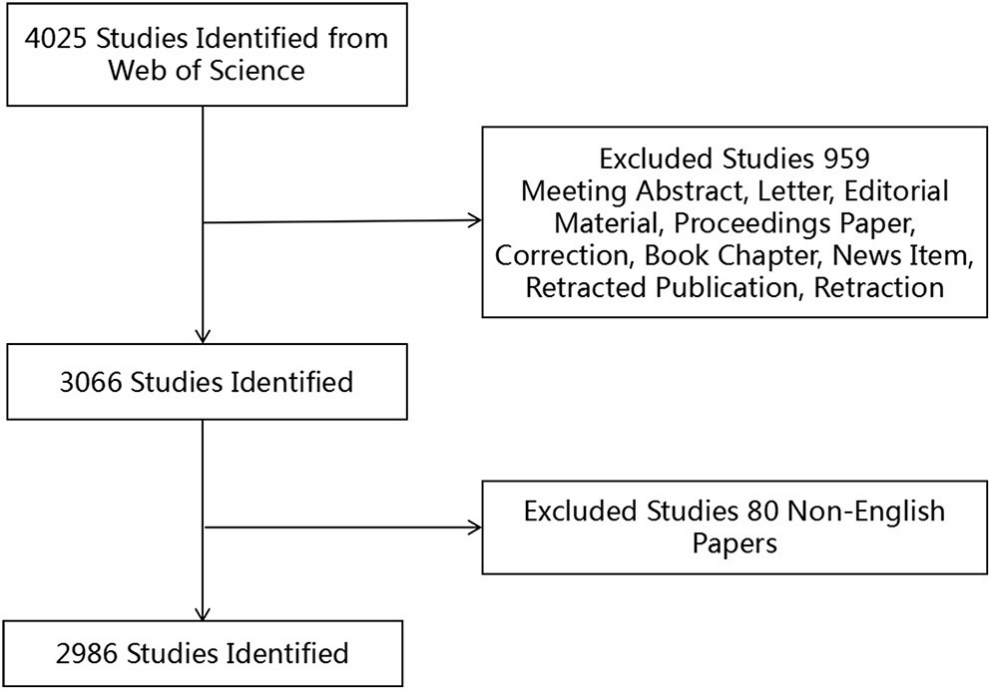
Flow chart of rTMS studies inclusion.

The inclusion criteria are shown in Figure 1. A record was considered relevant if “repetitive transcranial magnetic stimulation” or “rTMS” was found in its title, abstract, or keywords. Only articles and reviews were included; other document types, such as meeting abstracts and letters, were excluded. In addition, the publication language was restricted to English, and no species limitations were set. A total of 2,986 records published during the period 2009–2018 met the inclusion criteria.

### Analytical Methods

WoS provides detailed features of publications, such as number of papers, citations, citations per paper, essential science indicator (ESI) top papers, and Hirsch index (H-index). The number of papers reflects the research productivity. Citations measure the overall impact of the scientific output of a researcher, while the number of citations per paper measures the average impact. ESI top papers represent the most cited papers, including the hot papers and highly cited papers over the last 2 and 10 years, respectively (Fitzpatrick, 2005; Fu et al., 2011). The H-index is defined that a scientist has published h papers that have each been cited at least h times (if the H-index of a given individual is 10, this means that he published at least 10 papers and each of these 10 papers was cited at least 10 times) (Hirsch, 2005; McLean, 2019; Wang et al., 2019). The H-index evaluates the broad impact of the cumulative scientific publications of an author or country (Alonso et al., 2009; Bornmann and Daniel, 2009; da Silva and Dobranszki, 2018). Finally, impact factor (IF), according to Journal Citation Reports (2019), indicates the impact of journals.

CiteSpace V, a visual analytic system, is a good option for performing bibliometric analysis (Chen, 2004, 2006; Synnestvedt et al., 2005; Chen et al., 2012; Miao et al., 2017). CiteSpace V was used to perform co-citation analysis on authors, and synthesize and visualize the collaborations between countries into a network map which consists of a series of points and lines. In the network map, a point represents a country and a line between two points represents the cooperation relationship (Zheng and Wang, 2019). A wider line indicates a stronger relationship. More importantly, CiteSpace V can help detect the keywords and references with citation bursts. A citation burst has two characteristics, namely, strength and duration (Chen, 2006; Chen et al., 2012). A citation burst indicates increased attention to the underlying work over a certain period of time, which is a key indicator for determining emerging trends (Chen et al., 2014; Liang et al., 2017; Miao et al., 2017). GraphPad Prism 8, which has the basic functions of curve fitting and chart display of biological mathematical statistics, was applied to perform linear regression analysis and analyze the time trend of annual publication outputs.

## Results

### Publication Outputs and Time Trend

**Figure 2:**
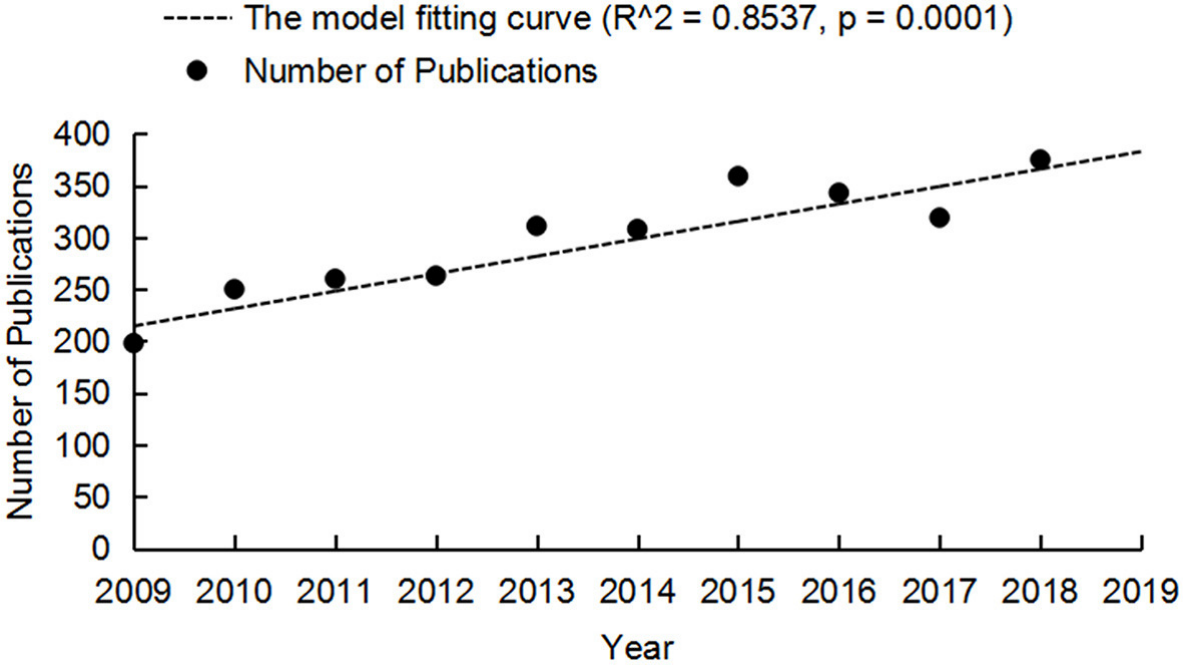
Annual publication outputs and the model fitting curve of time trend of rTMS publications.

A total of 2,986 publications were included in the analysis. The distribution and time trend of annual publication outputs at different time stages are shown in Figure 2. The overall trend is positive, and the publication output was 198 references in 2009 and 375 references in 2018. The time trend of publications indicated a significant correlation (R^2^ = 0.8537, *p* = 0.0001) between the annual publication outputs and the publication years in the last 10 years.

### Distribution by Journal

The 2,986 publications related to rTMS research were published in 565 scholarly journals. Amongst the top 20 journals shown in Table 1, the average IF was 3.660 (median 3.339, range 1.839–6.919). The journal *Brain Stimulation* with IF, 2018 = 6.919, published the most number of publications on rTMS research (199) and was cited 4,566 times, followed by *PLoS One* (publications, 89; IF, 2018 = 2.776; citations, 1269), *Clinical Neurophysiology* (publications, 66; IF, 2018 = 3.675; citations, 4,000), and *Neuropsychologia* (publications, 61; IF, 2018 = 2.872; citations, 1,015). *Clinical Neurophysiology* revealed the largest number of citations per paper published (60.61).

**Table 1:** The top 20 journals that published articles on rTMS research.

**Rank**	**Journal title**	**Count**	**IF 2018**	**Citations WoS**	**Citations per paper**	**Country**
1	*Brain Stimulation*	199	6.919	4,566	22.94	USA
2	*PLoS One*	89	2.776	1,269	14.26	USA
3	*Clinical Neurophysiology*	66	3.675	4,000	60.61	Ireland
4	*Neuropsychologia*	61	2.872	1,015	16.64	England
5	*Frontiers in Human Neuroscience*	56	2.870	986	17.61	Switzerland
6	*Neuroscience Letters*	51	2.173	542	10.63	Netherlands
7	*Restorative Neurology and Neuroscience*	50	1.839	761	15.22	Netherlands
8	*Journal of Ect*	49	2.280	492	10.04	USA
9	*Journal of Affective Disorders*	47	4.084	799	17.00	Netherlands
10	*Cerebral Cortex*	44	5.437	1,762	40.05	USA
11	*Neuroimage*	44	5.812	1,157	26.3	USA
12	*Journal of Neuroscience*	42	6.074	2,256	53.71	USA
13	*Psychiatry Research*	36	2.208	507	14.08	Netherlands
14	*European Journal of Neuroscience*	35	2.784	671	19.17	England
15	*Cortex*	30	4.275	685	22.83	Italy
16	*Experimental Brain Research*	30	1.878	791	26.37	Germany
17	*Frontiers in Neuroscience*	25	3.648	92	3.68	Switzerland
18	*Scientific Reports*	25	4.011	82	3.28	England
19	*Human Brain Mapping*	24	4.554	620	25.83	USA
20	*Journal of Cognitive Neuroscience*	24	3.029	556	23.17	USA

*IF, impact factor; WoS, Web of Science*.

### Distribution by Country and Institution

**Figure 3:**
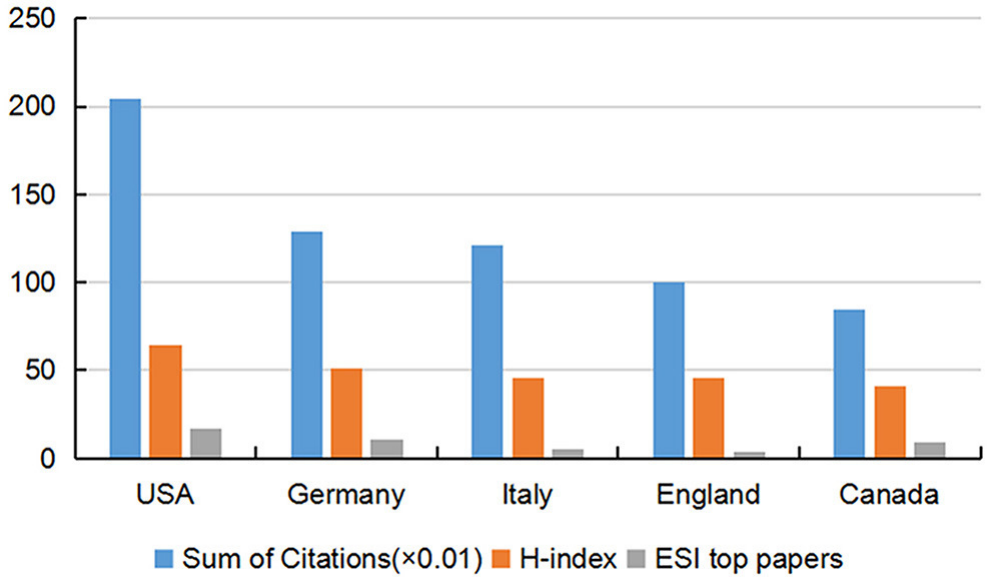
Citation counts (×0.01), H-index, and ESI top papers in the top five countries. ESI, essential science indicators; H-index, Hirsch index.

**Figure 4:**
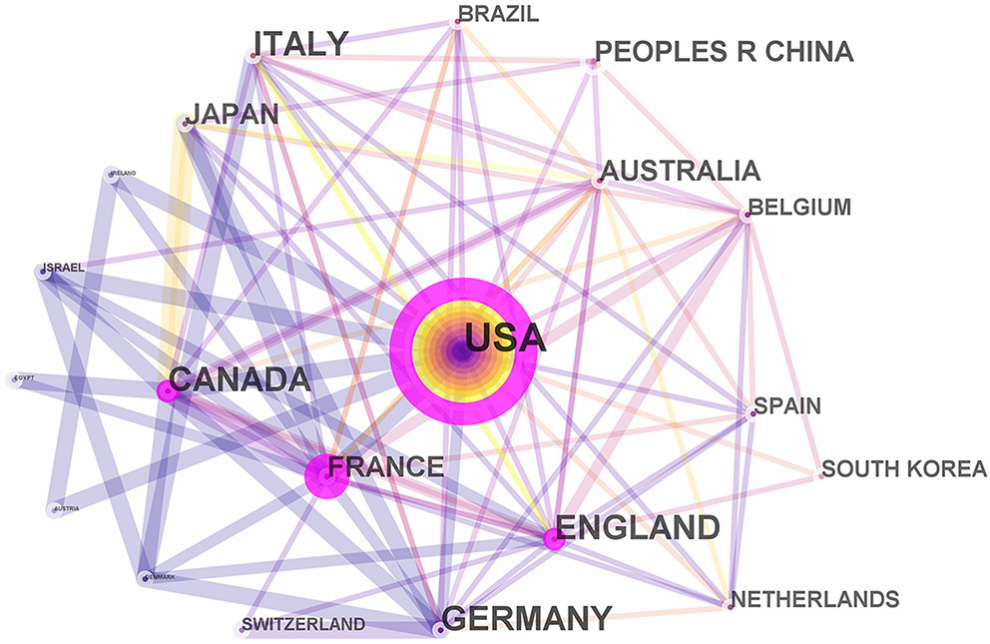
Network map of countries/territories engaged in rTMS research. In the network map, a point represents a country and a line between two points represents the cooperation relationship. A wider line indicates a stronger relationship.

All publications were distributed amongst 43 countries or regions. Amongst the 10 countries shown in Table 2, the USA had the largest number of published papers (764), citations (20,469) and ESI top papers (17) and the highest value of H-index (64). England revealed the largest number of citations per paper (33.91). Figure 3 provides an intuitive comparison of the citations, H-indices and ESI top papers of the top five countries publishing rTMS-related research, and the collaboration network amongst countries/territories is shown in Figure 4. Amongst the 2,986 publications included in this study, 22.67% were published by the top 10 most prolific institutions. University of Toronto (127) ranked first in the number of publications, followed by Harvard University (112), University College London (83), and Ghent University (58), as presented in Table 3.

**Table 2:** The top 10 countries of origin of papers in the rTMS research.

**Rank**	**Country**	**Count**	**Citations WoS**	**Citations per paper**	**H-index**	ESI top paper^*^
1	USA	764	20,469	26.79	64	17
2	Germany	414	12,870	31.09	51	11
3	Italy	411	12,122	29.49	46	5
4	England	296	10,037	33.91	46	4
5	Canada	273	8,477	31.05	41	9
6	China	266	2,712	10.20	28	1
7	France	213	7,266	34.11	37	6
8	Australia	208	4,947	23.78	37	3
9	Japan	145	4,641	32.01	28	3
10	South Korea	128	1,253	9.79	19	0

ESI, essential science indicators; H-index, Hirsch index; WoS, Web of Science.

* *There were a total of 33 ESI top papers*.

**Table 3:** The top 10 institutions contributed to publications on rTMS research.

**Rank**	**Institution**	**Count**	**Rank**	**Institution**	**Count**
1	University of Toronto	127	6	University of São Paulo	52
2	Harvard University	112	7	McGill University	50
3	University College London	83	8	Beth Israel Deaconess Medical Center	49
4	Ghent University	58	9	University of Regensburg	48
5	Monash University	52	10	Center for Addiction and Mental Health	46

### Distribution by Author

Over 9,600 authors contributed to the total output of rTMS research. The publication count in Table 4 reveals that Daskalakis ZJ published 81 papers, ranking first in terms of number of publications, followed by author A (78 publications), author B (65 publications), and Baeken C (51 publications). In terms of co-citation counts, Rossi S (817 citations) ranked first as the most co-cited author, followed by author B (594 citations), Wassermann EM (574 citations), and author C (518 citations).

**Table 4:** The top 10 authors and co-cited authors in rTMS research.

**Rank**	**Author**	**Count**	**Co-cited author**	**Count**
1	Daskalakis ZJ	81	Rossi S	817
2	Pascual-Leone A	78	Fitzgerald PB	594
3	Fitzgerald PB	65	Wassermann EM	574
4	Baeken C	51	George MS	518
5	George MS	46	Fregni F	477
6	Langguth B	46	Huang YZ	449
7	Zangen A	42	Lefaucheur JP	426
8	Lefaucheur JP	39	Chen R	421
9	Rothwell JC	39	Pascualleone A	415
10	Fregni F	38	Rossini PM	327

### Analysis of References

The evolution of a knowledge domain can be indicated by references with citation bursts (Synnestvedt et al., 2005; Chen et al., 2014). Table 5 shows the references with the strongest citation bursts during the period 2009–2018. Amongst them, citation bursts by the end of 2018 were led by author D's article published in 2014, which had the strongest burst (71.8868), followed by Rossini et al. (2015) and Berlim et al. (2014).

**Table 5:** References with the strongest citation bursts on rTMS research.

**References**	**Year**	**Strength**	**Begin**	**End**	**2009–2018**
Gershon et al. (2003)	2003	14.0082	2009	2011	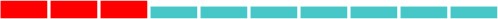
Robertson et al. (2003)	2003	6.5016	2009	2011	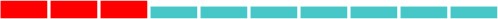
Huang et al. (2005)	2005	42.1427	2009	2013	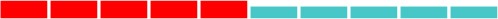
Strafella et al. (2003)	2003	6.9683	2009	2011	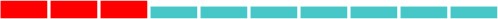
Siebner and Rothwell (2003)	2003	14.4801	2009	2011	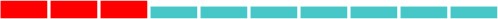
Siebner et al. (2004)	2004	14.9524	2009	2011	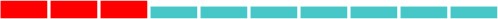
Fitzgerald et al. (2006a)	2006	13.4706	2010	2013	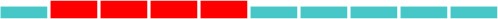
Iyer et al. (2003)	2003	11.6128	2010	2011	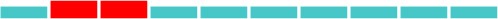
Herwig et al. (2007)	2007	11.6128	2010	2011	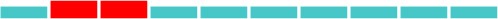
Gross et al. (2007)	2007	12.2034	2010	2013	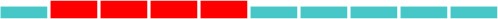
Herwig et al. (2003)	2003	13.0778	2010	2011	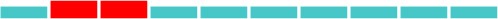
Mansur et al. (2005)	2005	14.0562	2010	2011	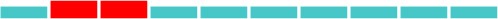
Naeser et al. (2005)	2005	12.6206	2011	2012	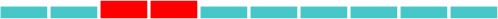
Fitzgerald et al. (2006b)	2006	18.3944	2011	2014	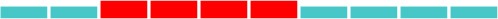
Oreardon et al. (2007)	2007	4.3588	2011	2015	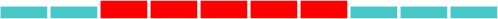
Fregni et al. (2006)	2006	10.2826	2012	2014	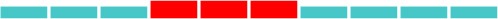
Lam et al. (2008)	2008	13.4243	2012	2013	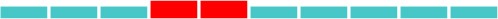
Hallett (2007)	2007	7.2548	2012	2015	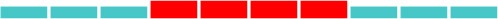
Schutter (2009)	2009	2.4316	2012	2013	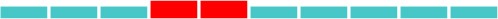
George and Post (2011)	2011	11.8953	2013	2014	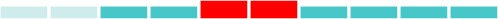
Pell et al. (2011)	2011	14.6987	2013	2016	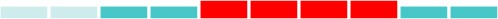
Ziemann et al. (2008)	2008	11.8953	2013	2014	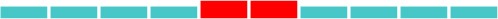
Huang et al. (2007)	2007	9.4852	2014	2015	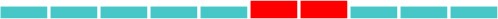
Hamada et al. (2013)	**2013**	**13.524**	**2014**	**2018**	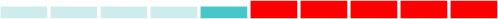
Cheeran et al. (2008)	2008	12.7504	2014	2015	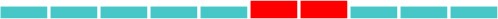
George et al. (2013)	2013	11.2585	2014	2015	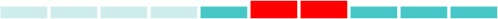
Ridding and Rothwell (2007)	2007	3.7702	2014	2015	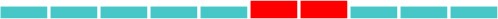
Fox et al. (2012)	2012	7.4318	2015	2016	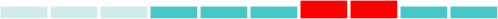
Ridding and Ziemann (2010)	2010	10.7746	2015	2018	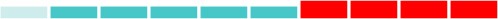
Berlim et al. (2014)	**2014**	**20.5935**	**2015**	**2018**	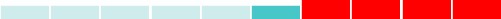
Cho and Strafella (2009)	2009	12.7212	2016	2018	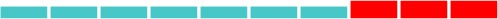
Deng et al. (2013)	2013	8.4915	2016	2018	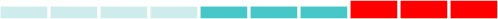
Rossini et al. (2015)	**2015**	**28.954**	**2016**	**2018**	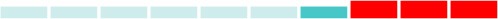
Hsu et al. (2012)	2012	9.7654	2016	2018	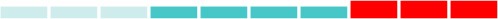
Fitzgerald et al. (2009)	2009	11.2418	2016	2018	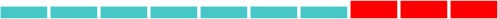
Guse et al. (2010)	2010	8.6631	2016	2018	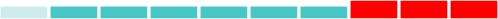
Berlim et al. (2013)	2013	12.2245	2016	2018	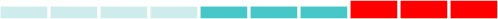
Lefaucheur et al. (2014)	**2014**	**71.8868**	**2016**	**2018**	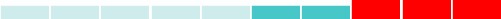
Bakker et al. (2015)	**2015**	**14.4765**	**2016**	**2018**	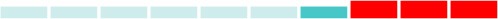
Gersner et al. (2011)	2011	9.4134	2016	2018	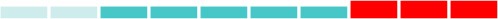

*The red bars mean the references cited frequently; the green bars mean the references cited infrequently. A greater strength indicates a higher frequency of citation. The references in bold were reviewed in this study*.

### Analysis of Keywords

Burst keywords can also be identified as indicators of emerging trends (Chen et al., 2014). Table 6 presents keywords with the strongest citation bursts in this field. The most recent burst keywords were “spinal cord injury,” “sham-controlled trial,” “recovery,” and “functional connectivity.”

**Table 6:** Keywords with the strongest citation bursts of publications on rTMS research.

**Keywords**	**Year**	**Strength**	**Begin**	**End**	**2009–2018**
Activation	2009	10.9509	2009	2014	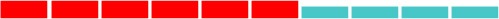
Premotor cortex	2009	8.197	2009	2010	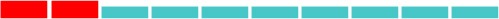
Perception	2009	7.4775	2009	2011	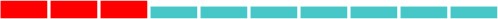
Cortical plasticity	2009	7.346	2009	2011	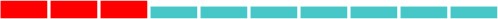
Human	2009	6.2637	2009	2011	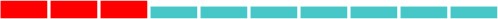
Synaptic plasticity	2009	5.9433	2009	2010	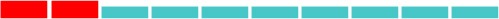
Working memory	2009	3.9615	2009	2013	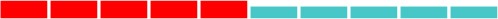
Paired associative stimulation	2009	3.8581	2009	2010	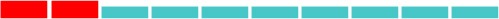
Corticospinal excitability	2009	8.7584	2010	2011	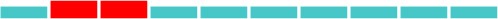
Intracortical inhibition	2009	8.5027	2010	2011	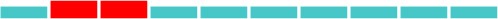
Cerebral blood flow	2009	8.0015	2010	2011	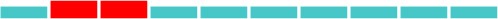
Treatment	2009	7.5005	2010	2011	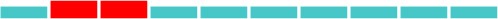
Positron emission tomography	2009	3.6267	2010	2015	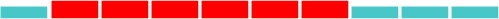
Aphasia	2009	10.4987	2011	2013	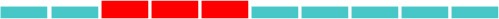
Tinnitus	2009	8.4556	2011	2012	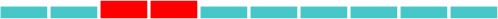
EEG	2009	5.6254	2011	2012	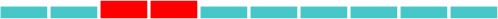
Mechanism	2009	3.662	2011	2012	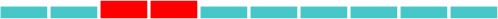
Primary motor cortex	2009	10.6895	2012	2014	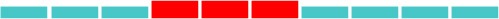
Language	2009	6.7568	2012	2016	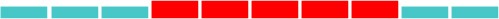
Human brain	2009	9.7966	2013	2014	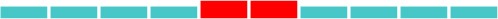
Therapy	2009	6.6686	2013	2014	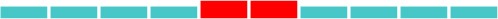
Neuropathic pain	2009	9.4064	2014	2015	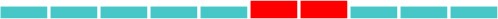
Inhibition	2009	8.5034	2014	2016	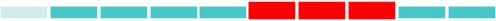
Spinal cord injury	2009	11.0035	2015	2018	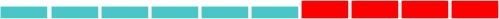
Sham controlled trial	2009	8.5157	2015	2018	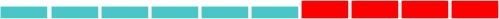
Alzheimers disease	2009	8.105	2015	2016	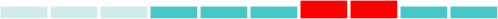
Recovery	2009	5.5658	2015	2018	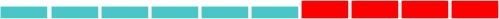
Functional connectivity	2009	4.0777	2015	2018	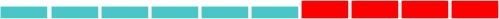

The red bars mean the keywords occurred frequently; the green bars mean the keywords occurred infrequently. A greater strength indicates a higher frequency of occurrence.

*EEG, electroencephalogram*.

## Discussion

### General Trends of rTMS From 2009 to 2018

rTMS has received great attention, and research related to the technique has been increasingly performed. It is reasonable to expect a promising future for rTMS based on analyzing the time trend of annual publication outputs.

Amongst the 20 top-performing journals, four journals, namely, *Brain Stimulation* (IF, 2018 = 6.919), *Journal of Neuroscience* (IF, 2018 = 6.074), *Neuroimage* (IF, 2018 = 5.812), and *Cerebral Cortex* (IF, 2018 = 5.437), had IF scores >5.000, and another seven journals had IF scores between 3.000 and 5.000. Approximately 19.09% (IF, 2018 > 5.000, 11.02%; 5.000 ≥ IF, 2018 ≥ 3.000, 8.07%) of the 2,986 publications involved were published in the top 20 journals with high IF (>3.000). In summary, it was challenging to publish papers related to rTMS in high-IF journals.

Amongst the top 10 countries, nine are developed countries and only one (i.e., China) is a developing country. From this point of view, there was still a wide gap between developed and developing countries in this filed. Although France revealed the largest number of citations per paper (74.6) amongst the top 10 countries publishing rTMS-related research, the USA ranked first in terms of publication count (764), citation count (20,469), and H-index (64). Moreover, the USA had more than half of the ESI top papers (17, 51.52%), which were hot papers and highly cited papers (Fitzpatrick, 2005; Fu et al., 2011). Therefore, the USA is the leading country in terms of the overall influence in this area.

Information on influential authors can help researchers identify potential collaborators. Authors A, B, C, D, and E were the most prolific and influential authors in this field, as determined by a comprehensive analysis of numbers of publications and co-citations. Author A suggested that rTMS of the left dorsolateral prefrontal cortex may be a potential treatment for depression (Pascual-Leone et al., 1996). Author B studied the neurobiological mechanisms of the antidepressant effects of rTMS and explored the use of rTMS for depression (Fitzgerald et al., 2006b; Arns et al., 2012; Fitzgerald and Daskalakis, 2012; Noda et al., 2015; Silverstein et al., 2015). Author C confirmed that daily left prefrontal rTMS is safe and effective for treating major depression (MD) (George et al., 2010). Author D showed extensive experience in treating neuropathic pain with rTMS (Lefaucheur, 2006; Lefaucheur et al., 2008b, 2012). Author E studied the effects of non-invasive brain stimulation, including rTMS and transcranial direct current stimulation on psychiatric disorders, pain, and neurological disorders (Lefaucheur et al., 2008a; Miniussi et al., 2008; Zaghi et al., 2009; Brunoni and Fregni, 2011).

### Emerging Trends of rTMS

The evolution of a knowledge domain can be reflected by keywords or references with citation bursts (Fitzpatrick, 2005; Chen, 2006). Therefore, this section analyzes keywords or references showing remarkable bursts by the end of 2018 to reveal emerging trends and provide directions for future research.

#### Keywords as Indicators of Emerging Trends

Burst keywords are considered indicators of emerging trends. Four emerging trends in rTMS research were determined according to the most recent keyword bursts; these keywords are listed as follows:

- Spinal cord injury (SCI): rTMS has emerged as a promising therapeutic technique for SCI patients (de Araújo et al., 2017; Nardone et al., 2017), and the technique has been applied to alleviate some of the main consequences of SCI, including sensory and motor function impairments, spasticity, and neuropathic pain (Tazoe and Perez, 2015; Gunduz et al., 2017). rTMS has also been used in animal experiments on SCI. For example, Krishnan et al. (2019) tested whether rTMS is effective in promoting plasticity and rehabilitation in a rat model of SCI, and their results suggested that rTMS can be used as an early intervention strategy; however, its efficacy and safety in clinical application should be further tested.
- Sham controlled trial: The type of stimulation is the key point of a sham-controlled trial. Although many studies have included sham-stimulation as control, realistic sham stimulation cannot be guaranteed in studies today (Mennemeier et al., 2009; Lefaucheur et al., 2014). High-quality sham-controlled trials are needed to design a completely blind research. As a preferential option for realistic sham trials, concomitant electrical skin stimulation may be superior to coil angulation and first-generation sham coils (Hosomi et al., 2013; Berlim et al., 2014; Lefaucheur et al., 2014).
- Recovery: rTMS has a positive impact on functional recovery, such as limb motor recovery in stroke patients; however, optimal rTMS parameters and high-quality evidence require further research (Pollock et al., 2014; Boonzaier et al., 2018; Yang et al., 2018; Xiang et al., 2019).
- Functional connectivity: Numerous neuropsychiatric conditions are reportedly coupled with network disturbances (Bassett and Bullmore, 2006, 2017; Grefkes and Fink, 2009, 2011; Frantzidis et al., 2014). As a means of modulating cerebral networks, rTMS not only interferes with the target cortex but also with those distant and interconnected areas, thereby restoring or increasing functional connectivity (Grefkes and Fink, 2009). Future studies on functional connectivity may facilitate new insights into the pathophysiology of neuropsychiatric conditions and optimize therapeutic strategies of rTMS (Grefkes and Fink, 2011; Krishnan et al., 2019; Kumru et al., 2019; Xiang et al., 2019).

### References With Strong Citation Bursts

References with citation bursts constitute an intellectual base, providing a better understanding of the trends of specific research fields (Fitzpatrick, 2005; Synnestvedt et al., 2005). Instead of discussing all the references with the strongest citation bursts, the following discussions will focus on the top five references by the end of 2018, which are shown in bold in Table 5.

As shown in Table 5, a paper by Lefaucheur et al. (2014) revealed the strongest burst by the end of 2018. In this paper, a group of European experts set up evidence-based guidelines on the clinical applications of rTMS in the neurological, ENT (ear, nose, and throat) and psychiatric domains. They also recommended that future technical developments of rTMS concentrate on the creation of new coil shapes and magnetic field geometries and progress of neuronavigation, especially combined with functional imaging and high-resolution EEG, for individualized rTMS treatment.

Rossini et al. (2015) revealed the second strongest citation burst by the end of 2018. The authors updated basic guidelines for clinical application and research on non-invasive stimulation in neuroscience and listed a number of unresolved issues. For example, they described the therapeutic applications of rTMS in depression and neuropathic pain through paradigmatic examples.

The next paper is Berlim et al. (2014), which represented the first meta-analysis to study response, remission, and dropout rates following HF-rTMS for MD. Some practical advice for future studies on rTMS for MD were also proposed. For instance, the authors recommended verification of the clinical utility of the targeted alternative brain region of HF-rTMS for MD.

Bakker et al. (2015) ranked fourth amongst the strongest citation bursts by the end of 2018. In this study, the authors observed 185 depression cases and found that intermittent theta burst stimulation (iTBS) and 5-fold longer 10 Hz protocols were comparable in terms of safety, tolerability and efficacy for dorsomedial prefrontal rTMS (30 min, 10 Hz vs. 6 min iTBS). Continuation of randomized trials of 10 Hz and iTBS in future work is recommended.

The burst duration in the study of Hamada et al. (2013) lasted 4 years beginning in 2014. In light of the individual variability existing in the after-effects of rTMS, Hamada et al. examined the effects of rTMS in 56 healthy subjects and provided evidence that individual variations in response to rTMS protocols are due to the neuronal networks activated by each TMS pulse.

To the best our knowledge, this study is the first to assess the trends of rTMS on the basis of literature published from 2009 to 2018 through a bibliometric approach. The study provides new insights for clinical and scientific growth and analyzes various aspects of published works. Nevertheless, this work has some limitations. The topic search was only conducted in SCI-EXPANDED of WoS and did not include other databases, such as PubMed and Scopus. Besides, non-English publications, which were few in number and may not change the conclusions, were excluded during retrieval. This study focuses on quantitative analysis but less qualitative analysis.

In conclusion, this study may help investigators discover the publication patterns and emerging trends of rTMS from 2009 to 2018, and presents reference values for the future research and applications of rTMS. The most influential author, institution, journal and country were author A, University of Toronto, *Brain Stimulation* and the USA, respectively. “Spinal cord injury,” “sham-controlled trial,” “recovery,” and “functional connectivity” may be the latest research frontiers. References with the most recent citation bursts, e.g., Lefaucheur et al. (2014), are worthy of attention and fundamental to emerging trends.

## Data Availability Statement

All datasets generated for this study are included in the article/supplementary material.

## Author Contributions

This study was conceptualized by K-YZ, G-YD, YL, and X-QW. K-YZ contributed to collecting data. Analyzing data and drafting the manuscript were by K-YZ and G-YD. K-YZ, G-YD, YL, and X-QW contributed to revising and approving the final version of the manuscript.

### Conflict of Interest

The authors declare that the research was conducted in the absence of any commercial or financial relationships that could be construed as a potential conflict of interest.
